# Genetics, Transcriptomics and Meta-Taxonomics in Visceral Leishmaniasis

**DOI:** 10.3389/fcimb.2020.590888

**Published:** 2020-11-25

**Authors:** Jenefer M. Blackwell, Michaela Fakiola, Om Prakash Singh

**Affiliations:** ^1^ Telethon Kids Institute, The University of Western Australia, Nedlands, WA, Australia; ^2^ IFOM—The FIRC Institute of Molecular Oncology, Milan, Italy; ^3^ Department of Medicine, Institute of Medical Sciences, Banaras Hindu University, Varanasi, India; ^4^ Department of Biochemistry, Institute of Science, Banaras Hindu University, Varanasi, India

**Keywords:** leishmaniasis, omics, genome-wide association study, transcriptional profiling, meta-taxonomics

## Abstract

Visceral leishmaniasis (VL) caused by parasites of the *Leishmania donovani* complex can be fatal in susceptible individuals. Understanding the interactions between host and pathogen is one way to obtain leads to develop better drugs and for vaccine development. In recent years multiple omics-based approaches have assisted researchers to gain a more global picture of this interaction in leishmaniasis. Here we review results from studies using three omics-based approaches to study VL caused by *L. donovani* in India: (i) chip-based analysis of single nucleotide variants in the first genome-wide association study of host genetic risk factors for VL, followed by analysis of epitope binding to HLA DRB1 risk versus protective alleles; (ii) transcriptional profiling demonstrating pathways important in Amphotericin B treated compared to active VL cases, including demonstration that anti-interleukin-10 unleashes a storm of chemokines and cytokines in whole blood responses to soluble leishmania antigen in active cases; and (iii) a meta-taxonomic approach based on sequencing amplicons derived from regions of 16S ribosomal RNA (16S rRNA) and 18S rRNA genes that allowed us to determine composition of both prokaryotic and eukaryotic gut microflora in VL cases compared to endemic controls. Overall, our omics-based approaches demonstrate that global analyses of genetic risk factors, host responses to infection, and the interaction between host, parasite and the microbiome can point to the most critical factors that determine the outcome of infection

## Introduction

Visceral leishmaniasis (VL) caused by parasites of the *Leishmania donovani* complex can be fatal in susceptible individuals. The number of new cases of VL caused by *L. donovani* *sensu strictu* was 93,660 between 2014 and 2018, with most cases found in India, Sudan, South Sudan, Ethiopia and Somalia (WHO, 2020). Vaccines are not available for leishmaniasis and we still rely on old toxic drugs requiring impractical expensive modes of administration poorly designed for the communities within which they are required to work. Understanding the interactions between host and pathogen is one way to obtain leads to develop better drugs and for vaccine development.

Through classical immunological studies it is has been shown that a *Leishmania*-specific cellular immune response correlates with control of infection in asymptomatic individuals, which could be measured as proliferation of lymphocytes following antigen stimulation of peripheral blood mononuclear cells (PBMC) (Sacks et al., 1987). PBMC from active VL cases are unresponsive to soluble *Leishmania* antigen (SLA) in these assays (Sacks et al., 1987). However, in whole blood assays active VL cases are able to make antigen-specific interferon-γ (IFN-γ) and tumor necrosis factor (TNF) cytokine responses produced by CD4^+^ T cells (Singh et al., 2012; Kumar et al., 2014). These pro-inflammatory cytokines are crucial for activation of macrophages to kill *L. donovani* parasites (Murray et al., 1983; Roach et al., 1991). These findings suggest that in human VL unfavorable clinical outcome is not related to an intrinsic defect in Th1 response. Instead, it was hypothesized that immunosuppressive or mechanisms of immune evasion could contribute to the pathogenesis of VL. Further studies comparing active with cured patients suggest that there is a balance between the pro-inflammatory cytokines TNF and IFNγ, and anti-inflammatory interleukin-10 (IL-10), with significantly elevated circulating levels of IL-10 in active compared to cured cases (Nylen and Sacks, 2007).

In recent years omics-based approaches have been harnessed to gain a more global picture of the interaction between host and pathogen, including in leishmaniasis [e.g. (Novais et al., 2015; Kong et al., 2017; Ashwin et al., 2018)]. Here we review results from studies using three different omics-based approaches to study VL caused by *L. donovani* in India: (i) chip-based analysis of single nucleotide variants used to carry out the first genome-wide association study (GWAS) of VL to identify host genetic risk factors (Fakiola et al., 2013); (ii) chip-based transcriptional profiling used to compare active with cured cases (Fakiola et al., 2019), including the influence of anti-IL-10 on whole blood responses to soluble leishmania antigen (SLA) (Singh et al., 2020); and (iii) a meta-taxonomic approach based on sequencing amplicons derived from regions of 16S ribosomal RNA (16S rRNA) and 18S rRNA genes that allowed us to determine composition of both prokaryotic and eukaryotic gut microflora in VL cases compared to endemic controls (Lappan et al., 2019).

## First GWAS Identifies HLA DRB1 as Main Genetic Risk Factor for VL

**Figure 1:**
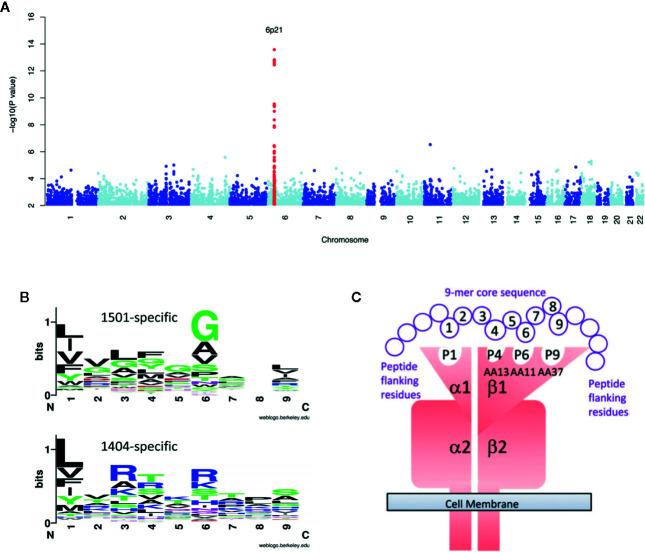
Provides a summary of studies which follow the story of how the first GWAS for VL led onto refined genetic and functional experiments that have given us a high resolution molecular insight into interactions between parasite antigenic epitopes and HLA Class II antigen-presenting cell molecules that drive the immune response towards either a protective or a disease-associated response. In **(A)** we show the original Manhattan plot resulting from meta-analysis of the GWAS data across 3 independent cohorts of VL caused by *L. donovani* in India and *L. infantum/chagasi* in Brazil (Fakiola et al., 2013). In **(B)** we show plots for sequence motifs that characterize 9-mer cores for epitopes binding preferentially to the protective DRB1*1501 compared to the DRB1*1401 risk allele (Singh et al., 2018). In these plots polar amino acids are shown in green (G, S, T, Y, C) or purple (Q, N), basic amino acids are blue (K, R, H), acidic amino acids are red (D, E), and hydrophobic amino acids are black (A, V, L, I, P, W, F, M). The size of the letter indicates the frequency with which the amino acid is found in this position of core 9-mers, and the overall peak height on the y-axis indicates the degree of conservation for specific epitopes at this location. From the plot we observe that there is more promiscuity (i.e. a greater range of different amino acids) and a higher frequency of basic amino acids in DRB1*1401-specific epitopes, compared with hydrophobic and polar amino acids in DRB1*1501-specific epitopes, at anchor residues P4 and P6. This was particularly important as these are the residues that interact with residues at DRB1 positions 11 and 13. In **(C)** we provide a model of how pathogen antigen epitopes bind to the DRA/DRB alpha/beta dimer. This shows the specific interaction between amino acids at positions 4 and 6 in the 9-mer cores of *Leishmania* antigenic epitopes that bind to pockets 4 and 6 created by amino acids at positions 11 and 13 in the DRB1 molecule. Elements of this figure are redrawn with permission from the original publications (Fakiola et al., 2013; Singh et al., 2018).

When complex segregation analysis of Brazilian families containing cases of VL caused by *L. chagasi* was performed it was found that genetic models for disease susceptibility provided the best fit to the data, with all single locus models providing a better fit than polygenic or multifactorial models (Peacock et al., 2001). Although it seemed unlikely that polymorphisms at a single locus could control a complex parasitic infectious disease, the first GWAS of VL (Fakiola et al., 2013) (**Figure 1A**) did find a single major peak of genome-wide significance (P_combined_=2.76 × 10^−17^; odds ratio=1.41; 95% confidence interval = 1.30–1.52 over three cohorts) at HLA-DR-DQ. Importantly, this association was replicated across three independent cohorts that crossed geographical continents and etiological parasite species, *L. infantum/chagasi* in Brazil and *L. donovani* in India. The results of this GWAS thus demonstrated that the strongest genetic risk factor for VL was at the core of generating a CD4+ T cell response. Based on these findings, further genetic and functional studies focused on determining the molecular interaction between antigen presentation by HLA class II molecules and antigenic molecules of the parasite. By undertaking more refined mapping it was possible to demonstrate that HLA-DRB1*1501 was the most protective risk allele, while DRB1*1404/DRB1*1301 were the most significant risk alleles (Singh et al., 2018). Furthermore, we demonstrated that specific residues at amino acid positions 11 and 13 were unique to protective alleles (Singh et al., 2018). *In silico* and *in vitro* experiments were used to analyze epitopes that bind to risk versus protective DRB1 alleles. Epitopes and binding affinities were mapped across 49 *Leishmania* vaccine candidates using NetMHCIIPan2.1 (Nielsen et al., 2010). Similar analysis was undertaken for protein antigens identified by capturing peptide epitopes from dendritic cells treated with *Leishmania* antigen (Singh et al., 2018). Details of these protein antigens are provided in the original manuscript. What was of particular interest was what we learned about epitopes within the proteins and how they bind to different DRB1 molecules. Across both data sets we demonstrated that sequence motifs for 9-mer core epitopes that bind to risk DRB1 alleles (*1404/*1301) showed greater peptide promiscuity compared to those that bind to protective (*1501) DRB1 alleles. We also observed (Singh et al., 2018) a higher frequency of basic AAs in DRB1*1404-/*1301-specific epitopes compared to hydrophobic and polar AAs in DRB1*1501-specific epitopes (**Figure 1B**) at anchor residues P4 and P6 which interact with residues at DRB1 position 11 and 13 (**Figure 1C**). Whole blood cytokine assays were then used to measure cytokine responses to 20-mer peptides that were synthesized to match the sequences of epitopes captured from risk versus protective alleles. Patients which had cured from VL made strong IFN-γ, TNF, and IL-10 responses, with peptides based on DRB1*1501-captured “protective” epitopes showing a bias towards IFN-γ over IL10 in response ratios compared to peptides based on DRB1*1301-captured “risk” epitopes. The initial GWAS study followed by more detailed fine mapping and functional experiments have thereby given us a high resolution molecular insight into interactions between parasite antigenic epitopes and HLA Class II antigen-presenting cell molecules that drive the immune response towards either a protective or a disease-associated response.

## Transcriptional Profiling of VL

Our interest in undertaking transcriptional profiling studies of VL in India was two-fold: (i) to obtain a global picture of changes in host response in infected but asymptomatic individuals, in active VL cases, and in treated VL cases, compared to healthy endemic controls; and (ii) to similarly profile the response of active cases to SLA in whole blood assays, and to determine what effect neutralizing anti-IL-10 would have on this response.

**Figure 2:**
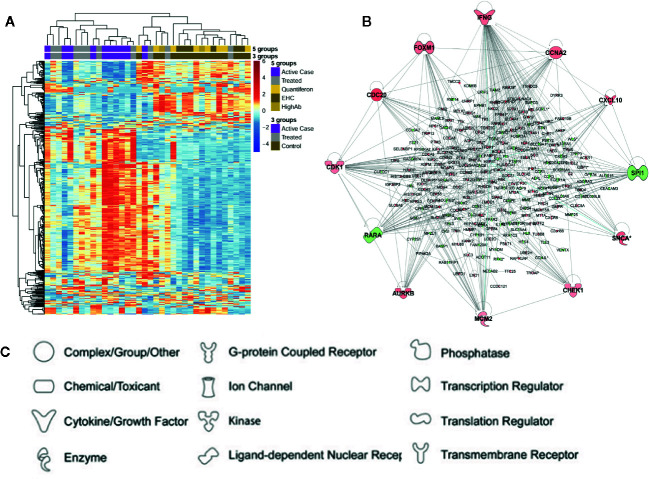
Summary of differential gene expression identified from transcriptional profiling of whole blood from active cases, asymptomatic individuals, and cases treated with Amphotericin B. In **(A)** we show a heatmap based on expression levels of the 500 most variable probes seen in one experiment (Fakiola et al., 2019). Columns represent individual samples and rows individual probes. Dendrograms based on hierarchical clustering are shown on the top and left side of the heatmap. Experimental groups are color-coded across the top of the heatmap. Keys show color coding for active (=case) and treated (=treated) cases, asymptomatics (=Quantiferon or HighAb positive individuals), and endemic healthy controls (=EHC). In **(B)** we show a gene network produced using Ingenuity Pathway Analysis for 254 DEGs identified in a comparison of active cases with healthy controls. Genes are represented by nodes. Genes in red have increased expression and genes in green have decreased expression when comparing active cases with healthy controls. The more intense the color the larger the fold change values. **(C)** Key to functional class of molecules encoded by genes at the nodes in **(B)**. Elements of this figure are redrawn with permission from the original publication (Fakiola et al., 2019).

For the first arm of this study we used chip-based transcriptomics to study expression profiles in whole blood from active VL cases, Amphotericin B treated VL cases, asymptomic individuals, and healthy unaffected endemic controls (Fakiola et al., 2019). Interestingly, no differentially expressed genes (DEGs) were identified when comparing a group of individuals who had asymptomatic infection as determined by high IgG or positive Quantiferon responses compared to healthy endemic controls who were negative by these assays (**Figure 2A**). Further analysis of the data therefore focused on comparisons between active cases and healthy controls, and between active cases and two groups of treated cases receiving either liposome-encapsulated or non-liposomal Amphotericin B. Results of particular interest included demonstration that active cases were enriched for gene sets associated with erythrocyte function and cell cycle compared to healthy controls, and that *IFNG* was the major hub gene (**Figure 2B**) when comparing active cases with healthy controls or with treated cases. Similarly, that interleukin signaling (IL-1/3/4/6/7/8) and chemokine (CXCL10/9/11) signaling were prominent pathways identified when comparing active cases with treated cases. One novel observation was that Aryl Hydrocarbon Receptor (AHR) signaling was a significant canonical pathway activated in VL cases. Also important was our demonstration of more effective cure, as determined by degree of return to “healthy endemic” baseline at day 30 post-treatment, when comparing a single dose of liposomal encapsulated amphotericin B with multi-dose non-liposomal amphotericin B treatment given over 30 days.

In association with *IFNG* as a hub gene multiple cytokine signaling pathways were perturbed. This included IFNG, IFNA, IL-1, IL-6, and TNF. Each of these cytokine pathways had DEG signatures that included CXCL10/11/9. This resulted in “pathogenesis of multiple sclerosis” (Sawcer et al., 2011) being identified as the top disease-related canonical pathway. This fits with the concept that a proinflammatory response contributes to disease pathology in active VL. In previous studies of spleen tissue and splenic macrophages in *L. donovani* infected hamsters (Kong et al., 2017), “Pathogenesis of multiple sclerosis” was also identified as a top canonical pathway. The authors of this hamster study also noted that high expression of IFN-γ was ineffective in activating macrophages to kill the parasite. The 3 chemokines CXCL10/11/9 are all induced by IFN-γ. They all bind to CXCR3 and play multiple roles in promoting T cell adhesion and in the chemoattraction of monocytes and macrophages, T cells, NK cells, and dendritic cells. In our study we also identified *GBP1* (Fakiola et al., 2019) as one of 10 top genes induced in active compared to treated VL cases. This gene encodes the interferon-γ-induced guanylate binding protein GBP1. Of interest, three genes identified in our study, *CXCL9*, *IFNG*, and *GBP1*, were also identified as part of a signature of 26 genes found to be upregulated in blood, spleen and liver across the course of experimental VL in mice (Ashwin et al., 2018) and may thus be strong biomarkers that could contribute to a signature for VL disease in humans. In this murine study, Cxcl9 and Gbp1 were also reported as hub genes from a STRING analysis (Ashwin et al., 2018).

Two observations made during our study were novel and interesting. One was the predominance of gene sets associated with erythroid cells and function that were enriched when comparing active VL cases and healthy controls. In a systematic review Goto and co-workers (Goto et al., 2017) had found that anemia has an overall prevalence >90% in VL. They noted that pathogenesis of anemia based on clinical observations includes the presence of anti-erythrocyte antibodies, dysfunction in erythropoiesis, and hemophagocytosis in spleen or bone marrow. They considered that, of these, hemophagocytosis was the most likely cause of anemia in VL cases (Goto et al., 2017). However, in our studies we saw differential regulation of gene sets associated with abnormal erythrocyte morphology, erythropoiesis, erythrocyte physiology, erythrocyte osmotic lysis, along with decreased hematocrit, spherocytosis and reticulocytosis. Hence, we concluded that the mechanisms associated with anemia in VL cases likely extend beyond just hemophagocytosis. Importantly, we propose that the erythoid-related gene sets identified by us could be used to monitor clinical cure. An example of this was that erythroid related genes had not returned to baseline following multi-dose Amphotericin B treatment compared to more effective treatment with single dose of liposomal Amphotericin B (Fakiola et al., 2019). Monitoring erythroid-related gene signals may this provide useful biomarkers for successful cure from VL.

The second novel observation of specific interest in our study was our demonstration that AHR signaling was the top canonical pathway when comparing active VL cases with either healthy controls or treated cases. AHR ligands have been shown to induce IL-10 secretion and inhibit IL-1β and IL-6 production in dendritic cells, and to promote IL-10 production and suppress IL-17 expression in CD4^+^ T cells (Wei et al., 2014a; Wei et al., 2014b; Wei et al., 2014c). IL-17 is also known to enhance recruitment and activation of neutrophils. Evidence from murine models (Goncalves-de-Albuquerque et al., 2017) shows roles for IL-17 and neutrophils in parasite clearance from liver and spleen, while both IL-10 and IL-17 cytokines are elevated in the serum of active VL patients (Duthie et al., 2014). IL-22 is another cytokine that is higher in *Leishmania* antigen stimulated peripheral blood mononuclear cells from active VL cases stimulated with *Leishmania* antigen compared to cases that have received treatment (Nateghi Rostami et al., 2016). Activation of AHR also inhibits inflammation by upregulating IL-22 (Monteleone et al., 2011). We therefore hypothesize that activation of AHR in VL cases could contribute to regulation of pro- and anti-inflammatory responses, both during active disease and in response to treatment. One interesting finding is that ligand-specific activation of AHR can lead to differentiation of inflammatory T helper 17 cells, or alternatively to modulation of regulatory T cells (Quintana et al., 2008). These authors showed that this ligand-specific activation could either exacerbate or suppress autoimmune disease. Hence we suggested (Fakiola et al., 2019) that such ligand-specific activation of AHR could be used as a therapeutic intervention to regulate the pathogenesis of VL.

**Figure 3:**
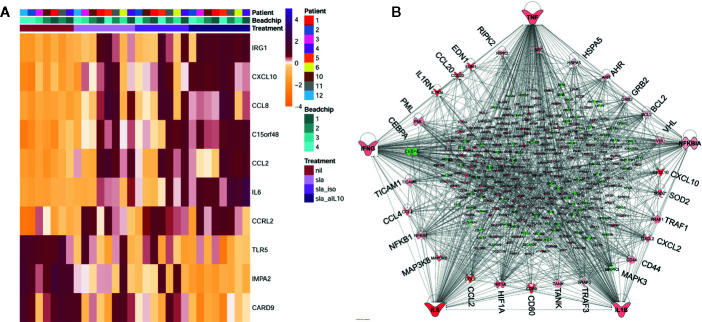
Summary of differential gene expression identified from transcriptional profiling of whole blood from active VL cases stimulated with SLA with/without anti-IL10 or isotype control. In **(A)** we show a heat map of z-score normalized gene expression values for probes representing the top 10 DEGs from the SLA plus anti-IL10 versus unstimulated blood analysis for all individuals across all experimental groups. Columns represent individuals and rows represent individual probes. Experimental groups are color-coded in the keys and upper part of the graph. **(B)** Ingenuity Pathway Analysis gene network for DEGs observed when whole blood was stimulated with SLA in the presence of anti-IL10. The network shows that 292 DEGs belong to a single network, with *TNF*, *IFNG*, *NFKBIA*, *IL6* and *IL1B* as the major hub genes in concert with a remarkable chemokine/cytokine storm. Genes annotated in red have increased expression, genes annotated in green have decreased expression, when comparing SLA plus anti-IL10 stimulated with unstimulated blood. The more intense the color the larger the fold change values. The key to molecular functions for genes at the nodes is as presented in **Figure 2C**. Elements of this figure are redrawn with permission from the original publication (Singh et al., 2020).

In the second arm of our transcriptional studies we used similar chip-based profiling in whole blood assays to study response to SLA in active VL cases with/without inclusion of neutralizing anti-IL-10 (**Figure 3A**). SLA stimulation alone identified only 28 DEGs, 17/28 in a single network with *TNF* at the hub and *IFNG* and *TREM1* as top upstream regulators. This response was muted compared to the response uncovered when anti-IL-10 antibody was included with SLA. The neutralization of endogenous IL-10 unleashed a storm of chemokine/cytokine responses, with key pro-inflammatory cytokines *TNF*, *IFNG*, *NFKBIA*, *IL6*, and *IL1B* as major hubs in a network of hundreds of DEGs (**Figure 3B**). *AHR* was also noted as a hub gene, suggesting that IL-10 modulates the Aryl Hydrocarbon Receptor signaling pathway which, as we observed above, is activated following Amphotericin B treatment in VL patients (Fakiola et al., 2019). *TREM1* signaling remained the top canonical pathway, with *TNF* and *IFNG* as top upstream regulators in the SLA plus anti-IL10 response. *TREM1*, the product of which is expressed on CD14^+^ monocytes, macrophages and neutrophils (Colonna, 2003), stands for the “triggering receptor expressed on myeloid cells 1”. It frequently acts together with TOLL-like and NOD-like pattern recognition receptors, acting to amplify inflammatory responses triggered by pathogens to stimulate the release of pro-inflammatory chemokines and cytokines.

One novel observation not noted in the comparison of unstimulated whole blood from active and treated VL patients (Fakiola et al., 2019) was identification of *ACOD1* as the top upregulated gene across all SLA stimulated groups. *ACOD1*, also known as *IRG1* (immune-responsive gene 1), encodes Aconitate Decarboxylase 1 which catalyzes production of the metabolite itaconic acid by decarboxylating *cis*-aconitate (Michelucci et al., 2013). Itaconic acid is known to inhibit isocitrate lyase, which is a key enzyme of the glyoxylate shunt pathway. The growth of isocitrate lyase-expressing bacteria such as *Salmonella enterica* and *Mycobacterium tuberculosis* is inhibited by itaconic acid (Michelucci et al., 2013), now recognized as a mammalian antimicrobial metabolite (Cordes et al., 2015). Although isocitrate lyase and malate synthase, which are the two key enzymes of the glyoxylate cycle, have not been annotated in *Leishmania* genomes, spectrophotometrically assayed isocitrate lyase activity has been reported (Hernandez-Chinea et al., 2017). Hence, it is unclear whether ACOD1 could contribute directly to anti-leishmanial activity through production of itaconic acid. Daniels et al. (Daniels et al., 2019) demonstrated that IRG1 led to antiviral activity through itaconate inhibition of succinate dehydrogenase, generating a metabolic state that suppressed Zika viral genome replication. Another role of ACOD1/IRG1 is as a negative regulator of TLR-mediated inflammatory innate responses in sepsis-associated immunosuppression (Li et al., 2013). IRG1 is highly upregulated in PBMC from septic patients, and suppresses TLR-triggered proinflammatory responses through elevated reactive oxygen species stimulating TNF-induced TNFAIP3 (A20; also seen as a top DEG in our study (Singh et al., 2020)) expression (Li et al., 2013). This compromises the ability of sepsis patients, who may survive the early uncontrolled over-activation of the innate pro-inflammatory response, to later deal with opportunistic co-infections. Clearly a balance must be achieved between stimulation of pro-inflammatory antimicrobial responses to control infection while avoiding hyperinflammation that can lead to organ failure and death. Our data provide valuable new knowledge that could inform the application of IL-10–directed immuno-therapeutics for the treatment of VL.

## Meta-Taxonomic Study of Prokaryotic and Eukaryotic Gut Microflora in VL

Recent interest has focused on the possible interaction between human hosts and their microbiome in determining the outcome of parasitic infections. For example, people who are at risk of VL are also at risk of other neglected tropical diseases (NTDs) such as soil transmitted helminths. Helminths of the intestine are very strong regulators of immune responses in their hosts and can ameliorate inflammatory diseases (Zaiss et al., 2015), sometimes mediated through cross-talk with gut microbiota. As a background to our study there was not much information on how *L. donovani* infections interact with other NTDs. In a study of *L. donovani* in mice with established *Schistosoma mansoni* infection it was shown (Hassan et al., 2006) that mice failed to control *L. donovani* even though they had had made a strong *L. donovani*-specific T helper 1 response. Similarly, when mice chronically infected with the intestinal nematode *Heligmosomoides polygyrus* were infected with *L. donovani* they had higher numbers of parasites in liver and spleen compared to worm free mice (Classon et al., 2019). A factor which limits large-scale analysis of pathogens in the gut is the time-consuming microscopic identification of parasites. Recent studies have shown that multiplex PCR for fecal DNA samples can be applied successfully in large-scale studies of specific helminth and protozoan gut pathogens (Llewellyn et al., 2016). However, this does not provide data on the influence these pathogens might have on the broader gut microflora. We therefore undertook a study to evaluate the use of amplicon-based sequencing of 16S ribosomal RNA (16S rRNA) gene regions to identify prokaryotic flora and 18S rRNA gene regions to identify eukaryotes using a meta-taxonomic approach (Lappan et al., 2019).

**Figure 4:**
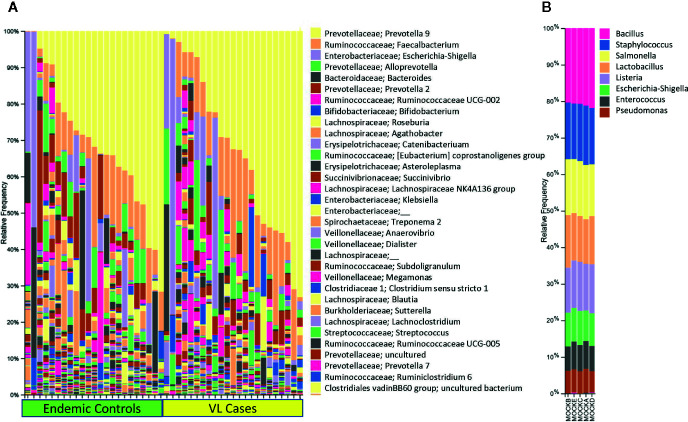
Shows 16S rRNA-determined prokaryotic gut microbial profiles in VL cases and endemic controls. In **(A)** we show bar plots that indicate the relative abundance of 16S rRNA taxa separated for VL cases and endemic control groups, each ordered by relative abundance of taxa color coded as per key shown on the right of the plot. In **(B)** we provide bar plots for prokaryotic mock control samples that were used in the experiment. Elements of this figure are redrawn with permission from the original publication (Lappan et al., 2019).

In this small scoping study we examined 16S rRNA and 18S rRNA profiles in 23 active cases of VL compared to 23 geographically matched healthy endemic controls from Bihar State India. Using QIIME software (Caporaso et al., 2010; Bolyen et al., 2018) to analyze the amplicon sequence libraries (**Figure 4**) the most abundant bacterial taxa identified across all fecal samples were *Prevotella* (37.1%), *Faecalibacterium* (11.3%), *Escherichia-Shigella* (9.1%), *Alloprevotella* (4.5%), *Bacteroides* (4.1%), *Ruminococcaceae* UCG-002 (1.6%), and *Bifidobacterium* (1.5%) (Lappan et al., 2019). It was of interest to compare these results to previous reports of the composition of gut microflora across India. For example, a study from urban and semi-urban districts in West India (including Mumbai) also found gut microflora dominated by *Prevotella* (34.7%), *Bacteroides* (15.2%), and *Faecalibacterium* (5.6%) (Kulkarni et al., 2019). Notable, however, were additional prominant species of *Megasphaera* (4.7%) and *Dialister* (3.9%) (Kulkarni et al., 2019) not seen in our data. *Prevotella* (4.5%) and *Megasphaera* (8.5%) were also dominant genera found in gut microflora in Delhi in the North and Pune in the West (Bhute et al., 2016), the authors of this study suggesting that these two genera were a distinctive feature of Indian gut microflora compared with western cultures. *Prevotella* was also the dominant genus in North-Central India where residents eat a predominantly plant-based diet compared to omnivorous populations in Southern India where more prominent associations with *Bacteroides*, *Ruminococcus*, and *Faecalibacterium* were observed (Dhakan et al., 2019). This was in concordance with previous classification of human gut microbiome enterotypes (Arumugam et al., 2011). North-Central populations were associated with enterotype-2 (73.5%) driven by *Prevotella* whereas in Southern India (54% enterotype-2) enterotype-1 (30.3%) driven by *Bacteroides* and enterotype-3 (16.1%) driven by *Ruminococcus* were also prevalent. In our study (Lappan et al., 2019), bacterial taxa (e.g. *Dialister*, *Megasphaera*, *Mitsuokella*, *Lactobacillus*) normally characteristic of Indian gut microbiomes (Bhute et al., 2016; Dhakan et al., 2019; Kulkarni et al., 2019) occurred at <1%. Hence this population from Bihar in North-East India did not fall within the criteria previously identified as being characteristic of Indian populations.

Our study was unique in identifying Eukaryotic taxa (Lappan et al., 2019). We observed *Blastocystis* (57.9%), *Dientamoeba* (12.1%), *Pentatrichomonas* (10.1%), *Entamoeba* (3.5%), Ascaridida (0.8%), Rhabditida (0.8%), and Cyclophyllidea (0.2%). The latter 3 helminth identifications were concordant with *Ascaris*, *Strongyloides*, and *Hymenolepis* identified by microscopy. The study was successful in demonstrating that eukaryotic gut flora and pathogens, including helminths, could be detected using 18S rRNA gene region sequencing. Our sample size was not sufficiently powered to test for statistical associations between VL and helminth infections, but other interesting observations were made relating to eukaryotic pathogens as outlined below.

**Figure 5:**
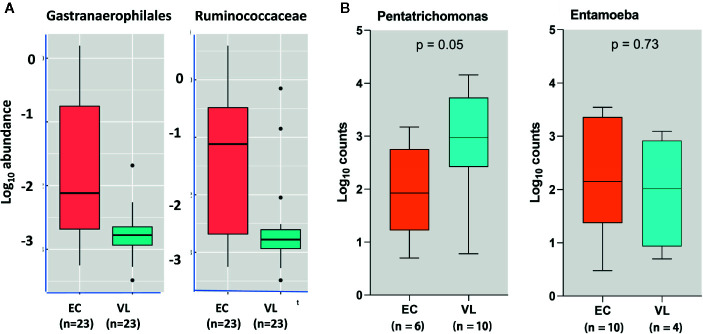
Taxon-specific differences in gut microbes observed in active VL cases compared to endemic controls (EC) using meta-taxonomics. In **(A)** we show box plots derived using a program called ANCOM that showed that EC have higher abundance of *Gastranaerophilales* and *Ruminococcaceae* prokaryotic taxa than VL cases. In **(B)** we show box plots that compare log_10_ counts for *Pentrichomona*s and *Entamoeba* in EC versus VL cases. From this plot we see that VL cases have higher counts for *Pentatrichomonas* but not for *Entamoeba*. Elements of this figure are redrawn with permission from the original publication (Lappan et al., 2019).

For both prokaryotic and eukaryotic gut flora, diversity indices that provide a global measure of differences in composition of gut flora, did not differ significantly according to disease status (i.e. VL case or healthy endemic control), or by age, sex, geographic subdistrict. That is, we did not find evidence for global differences or dysbiosis in eukaryotic or prokaryotic gut flora in comparing VL cases with endemic controls in this region of India. However, some taxon-specific associations were observed (Lappan et al., 2019) which we show here in **Figure 5**. Firstly, we observed that Ruminococcaceae UCG- 014 and Gastranaerophilales_uncultured bacterium were enriched in endemic controls compared to VL cases (**Figure 5A**). Secondly, we found that *Pentatrichomonas hominis* was more abundant in VL cases than in endemic controls, whereas the trend was reversed for *Entamoeba* (**Figure 5B**). When we looked at the cohort as a whole we found that high *Escherichia-Shigella* was associated with reduced bacterial diversity, whereas high *Blastocystis* was associated with high bacterial diversity and low *Escherichia-Shigella*. In individuals that had high *Blastocystis* counts, we observed low Bacteroidaceae and high *Clostridiales* vadin BB60. In contrast, those with low *Blastocystis* had high Bacteroidaceae and low *Clostridiales*. In the literature it is generally considered that *Bacteroides* are friendly commensals in the gut (Wexler, 2007). They are associated with fermentation of carbohydrates thus producing volatile fatty acids. These volatile fatty acids contribute to the daily energy requirement of the host (Hooper et al., 2002). In our study high *Blastocystis* seemed to put *Bacteroides* at a disadvantage. Hence, we hypothesized that *Blastocystis* was influencing gut flora homeostasis. Although high *Blastocystis* was negatively correlated with *Escherichia-Shigella* in our study, we concluded that it may contribute to overall dysbiosis of gut microflora and to the low prevalence of enterotype-1 normally characterized by high *Bacteroides*.

In brief, our meta-taxonomic analysis of gut microflora provides novel data on both prokaryotic and eukaryotic gut microflora for a population in India that is endemic for VL. We observed dysbiosis related to pathogenic prokaryotic and eukaryotic species that influenced global measures of bacterial diversity in the gut. We also observed some important associations between the eukaryotic pathogen *P. hominis* and VL. Our data provide a useful baseline upon which a broader analysis of pathogenic enteric microflora and their influence on gut microbial health and NTDs generally can be developed.

## Conclusions

Together our genetic and transcriptional profiling studies have provided important evidence to support the importance of antigen presentation to CD4+ T cells and the role of IFNγ in the pathogenesis of *L. donovani* infection. In a more recent GWAS for cutaneous leishmaniasis in Brazil (Castellucci et al., 2020) we were able to carry out integrated post-GWAS annotation that related genetic risk loci to public domain data on expression quantitative trait loci as well as disease-specific transcriptional data that allowed us to focus in on the most plausible genetic risk loci. Application of this type of integrated analysis might highlight additional genetic risk loci for VL that were not apparent in our meta-analysis of VL caused by *L. donovani* in India compared to *L. chagasi* in Brazil. Others have also demonstrated that polymorphisms in human genes can be associated with signatures of microbiomes, for example the esophageal microbiome that influences esophageal adenocarcinoma (Deshpande et al., 2018). Hence, while our separately undertaken omics-based approaches have demonstrated that global analyses of genetic risk factors, host responses to infection, and meta-taxanomic analysis of the microbiome can point to critical factors that determine the outcome of infection, a more integrated approach might yet be more fruitful in future studies.

## Author Contributions

JB prepared the manuscript and the original papers reviewed here. MF analyzed omics-based data and prepared some of the figures for incorporation in this review. OS carried out original field work and sample preparation. All authors contributed to the article and approved the submitted version.

## Funding

As reported in our primary publication, the studies that we have reviewed here were undertaken as part of an NIH Tropical Medicine Research Centre grant (Grant number U19 AI074321), with additional support from The Wellcome Trust (grant numbers 074196/Z/04/Z, 085475/B/08/Z and 085475/Z/08/Z). MF was supported by a Raine Visiting Fellowship to the University of Western Australia.

## Conflict of Interest

The authors declare that the research was conducted in the absence of any commercial or financial relationships that could be construed as a potential conflict of interest.
